# We still need to operate at night!

**DOI:** 10.1186/1749-7922-2-29

**Published:** 2007-10-31

**Authors:** Omar Faiz, Saswata Banerjee, Paris Tekkis, Savvas Papagrigoriadis, John Rennie, Andrew Leather

**Affiliations:** 1 Department of General Surgery, Kings College Hospital, Denmark Hill, London, UK; 2 Academic Surgical Unit, St Mary's Hospital, Paddington, London, UK

## Abstract

**Introduction:**

In the past the National Confidential Enquiry into Peri-operative deaths (NCEPOD) have advocated a reduction in non-essential night-time operating in NHS hospitals. In this study a retrospective analysis of the emergency general surgical operative workload at a London Teaching centre was performed.

**Methods:**

All general surgical and vascular emergency operations recorded prospectively on the theatre database between 1997 and 2004 were included in the study. Operations were categorised according to whether they commenced during the daytime(08:01–18:00 hours), evening(18:01–00:00 hours) or night-time(00:01–08:00 hours). The procedure type and grade of the participating surgical personnel were also recorded. Bivariate correlation was used to analyse changing trends in the emergency workload.

**Results:**

In total 5,316 emergency operations were performed over the study period. The numbers of daytime, evening and night-time emergency procedures performed were 2,963(55.7%), 1,832(34.5%), and 521(9.8%) respectively. Laparotomies and complex vascular procedures collectively accounted for half of all cases performed after midnight whereas they represented only 30% of the combined daytime and evening emergency workload. Thirty-two percent (n = 166) of all night-time operations were supervised or performed by a consultant surgeon. The annual volume of emergency cases performed increased significantly throughout the study period. Enhanced daytime (*r *= 0.741, p < 0.01) and evening (*r *= 0.548, p < 0.01) operating absorbed this increase in workload. There was no significant change in the absolute number of cases performed at night but the proportion of the emergency workload that took place after midnight decreased significantly throughout the study (*r *= -0.742, p < 0.01).

**Conclusion:**

A small but consistent volume of complex cases require emergency surgery after midnight. Provision of an emergency general surgical service must incorporate this need.

## Introduction

Over the last decade significant change has occurred to the provision of the emergency service in many hospitals. The principal factors underlying this change have been the influence of recommendations made by the 'National Confidential Enquiry into Peri-operative Deaths' (NCEPOD) [1,2] as well as a mandatory reduction in junior doctor working hours brought about by the 'European Working Time Directive' (EWTD) [3].

In 2003 NCEPOD repeated a comprehensive 'Who Operates When' (WOW) audit to investigate the provision of emergency surgical services in the United Kingdom [2]. The first audit of this kind was performed in 1997 and following that important recommendations were made[1]. More specifically, NCEPOD stated that emergency surgical patients should expect to be treated by trained personnel regardless of when they presented or required surgery. In consequence, recommendations were made to enhance senior-led service provision. To this end suggestions were made to increase the availability of dedicated daytime emergency lists and thereby move away from out-of-hours operating where possible.

The success of this initiative is evidenced by the results published in the 2003 WOW II report. More specifically when the 2003 data is compared to the 1997 WOW I data significant increases in consultant surgeon participation in emergency cases was observed during: daytime (41% vs. 28%), evening (21% vs.14%) and night-time (26% vs.11%) hours. Similarly, increased availability of scheduled daytime emergency operating sessions (63% vs. 51%) was also noted at participating Trusts over this period.

The aims of this study were to analyse the: volume, content and consultant supervision of the emergency general surgical operative workload at a London Teaching hospital and, in addition, to identify changing trends in the volume of the emergency workload.

## Materials and methods

A retrospective analysis of all emergency general surgical and vascular procedures performed at a London Teaching centre between April 1997 and April 2004 was performed.

### Data management

The data used in this study was acquired from e theatre database. At our centre an electronic database system (Surgiserver^© ^McKennon Systems) is used to capture theatre data prospectively. Recorded theatre data comprises: the type of procedure performed, the timing and duration of an operation as well as the key theatre personnel involved in a procedure. In this study historical theatre data was converted to Excel (^©^Microsoft) and SPSS (^©^SPSS Inc.) formats for data recoding and subsequent statistical analysis.

The time when anaesthesia was commenced was used as the procedure 'start time'. The latter was then recoded according to whether the procedure commenced during the: daytime (08:01–18:00 hours), evening (18:01–00:00 hours) or night-time (00:01–08:00 hours). In addition, surgical personnel that performed or participated in emergency procedures were recoded according to whether they were consultants or trainees.

### Statistics

Bivariate (Spearman's) correlation was used to analyse trend changes in the absolute numbers of emergency cases performed over time as well as changing trends in the proportion of total workload carried out during daytime, evening and night-time hours. Statistical significance was assumed where P < 0.05.

## Results

The study centre is a Teaching Hospital located in South East London. It serves a population of 225,000 people. Eight general surgical consultants and three vascular consultants participate in the provision of the emergency service. At the time that the study was conducted consultant surgeons and registrars worked an on-call system without any reprieve from elective commitments whilst on. In contrast, Senior House Officers were mostly free from elective duties whilst attending to emergencies. Time off the day after an on-call was not routinely afforded to surgical personnel following emergency nighttime operating.

In total 5,316 general surgical and vascular emergency operations were performed between April 1997 & April 2004. Fifty four percent of all procedures were performed on men (n = 2,871) and the median patient age was 44 years (range, 12–99 years).

### Timing of surgery

Overall 55.7% (n = 2,963) of all study procedures were carried out during daytime hours whilst a further 34.5% (n = 1,832) occurred during the evening and 9.8% (n = 521) took place after midnight.

### The content of the emergency workload

Three surgical procedures accounted for two-thirds of the total emergency workload. The latter operations comprised: appendicectomy (17.7%), incision and drainage of abscess (21.2%) and laparotomy (28.6%).

**Figure 1:**
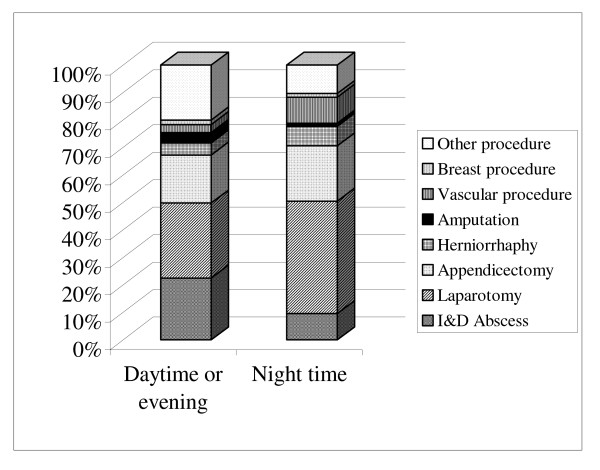
The operative content of the emergency workload (% of total) according to timing of surgery.

Comparison of the relative proportions of total workload carried out during daytime and night-time hours was performed (Figure 1). After midnight an increase in the proportion of the operative workload made up by urgent cases, such as complex vascular operations and laparotomies, was noted. Conversely, the proportion of total night-time workload made up by less urgent cases, such as abscess drainages, was lower than that observed during daytime and evening hours. According to the British Union Provident Association (BUPA) classification of procedures – operations categorized as 'Minor' or 'Intermediate' represented 28% and 12% of the total daytime and night time operating respectively. Conversely the proportion of procedures categorized as either 'Major', 'Major+' or 'Complex Major' was higher during the night (77%) when compared to the daytime workload (52%).

### Consultant surgeon participation in emergency operations

A consultant surgeon was present in 36.2% (n = 1,925) of all emergency general surgical cases recorded throughout the study. Consultant presence was greatest during daytime (38.2% cases) and evening hours (34.2% cases), but despite this, 31.9% of all nighttime cases were still supervised by a senior.

### Trends in the emergency workload

**Figure 2:**
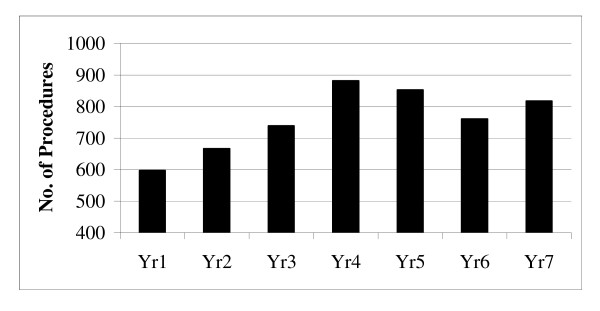
Annual volume of emergency general surgical procedures performed between 1997 and 2004.

**Figure 3:**
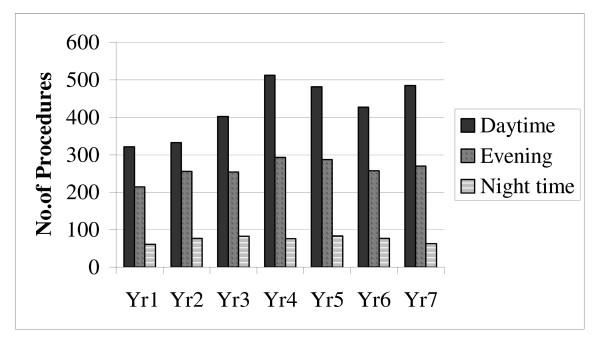
Annual volume of emergency general surgical procedures performed according to timing of surgery.

Throughout the study period an increase in the annual number of emergency general surgical operations performed at our centre was noted (Figure 2). Figure 3 illustrates the latter annual operative workload divided according to time of day category.

Statistical analysis confirmed significant increases in the absolute quantity of daytime (*r *= 0.741, p < 0.01) and evening (*r *= 0.548, p < 0.01) emergency procedures performed over time.

In contrast, the absolute annual numbers of operations performed after midnight remained static throughout the study period (*r *= -0.025, p=NS). However, as an increase in the annual total operative volume was observed throughout the study period – when the nighttime workload was considered as a proportion of the total emergency load – a significant decrease in night work was detected over time (*r *= -0.742, p = 0.01).

## Discussion

An increase in the volume of the emergency general surgical operative workload was observed at our centre over the study period. The closure of the Accident & Emergency department of another South East London hospital seven years ago has almost certainly contributed to this finding.

The definition of surgical procedures as per revised NCEPOD guidelines indicates an 'Immediate (A) life saving and (B) limb or organ saving' operation to be usually performed within minutes of decision to operate with simultaneous resuscitation. An 'urgent' operation is performed within 'hours' of decision to operate and normally after resuscitation [4]. Table 1 provides general guidelines for surgical procedures to be performed at 'night' as per revised NCEPOD classification based on the experience at our centre. It is important to state that individual clinical situations determine decisions regarding appropriate timing for surgery.

**Table 1:** Guidelines for surgical procedures (excluding obstetrics) to be performed at night as per revised NCEPOD classification

**Immediate**	**Vascular**	**Ruptured AAA**
	**Trauma**	Major trauma to thorax/abdomen with haemodynamic compromise
	**Urology**	Suspected testicular torsion
**Urgent**	**Abdomen**	Perforated viscus
		Penetrating abdominal injures
		Peritonitis
		Gastrointestinal haemorrhage with haemodynamic compromise
		Intestinal obstruction with possible bowel infarction
		Strangulated hernia
		Acute appendicitis (especially in children and elderly)
	**Vascular**	Critical limb ischaemia
	**Orthopaedics**	Fracture with major neurovascular deficit
		Compartment syndrome
		Compound fracture

In a multi-centre study carried out over a decade ago a consensus panel of surgeons determined that approximately one-third of emergency cases carried out at night could have safely been deferred to the following day [5]. Indeed, numerous studies have now corroborated that improved daytime emergency theatre access can lead to significant reductions in night-time operating [6-8]. These factors, along with the resultant enhanced consultant supervision that accompanies daytime operating, previously led the 1997 NCEPOD report authors to urge for improved access to scheduled emergency theatre lists. Our study suggests that the majority of the increased emergency workload experienced by our department was absorbed through enhanced daytime operating. In turn, a reduction in the proportion of emergency operating that was carried out at night was also achieved throughout the study period. In addition, the consultant supervision of the 'out-of-hours' emergency work carried out at our centre compares favourably with the national 2003 NCEPOD report figures. Importantly, no set protocols or guidelines were used in the study centre to establish the grounds for consultant attendance at 'out-of hours' procedures. The decision to carry out an operation at night was generally taken by the on-call surgical registrar and, for the more severe cases requiring complex surgery, this decision was generally discussed with the consultant on-call who would in turn decide on whether to attend. It must be stated however that concerns have been raised that the recent increase in consultant participation in the emergency service has resulted in decreased operative experience amongst junior staff [9].

Despite the reduction in the proportion of emergency cases that required 'out-of-hours' operating, at our centre a small consistent volume of cases required an operation at night. Furthermore, analysis of the operative case-mix of the night-time workload suggests that there was an increased proportion of urgent cases operated after midnight. The latter provides indirect evidence that the timing of these procedures was influenced, at least to some extent, by clinical urgency. It has been suggested by various investigators that short delays to some emergency operations, such as appendicectomies, does not result in undue adverse consequences [10]. In our study some postponement of overnight cases almost certainly took place. This is suggested by the lower proportion of the nighttime case-mix comprising less urgent procedures such as abscess drainages. It must be stated clearly however that not all surgeons accept that operative delays, incurred to emergency patients solely on the basis of their timing of presentation, are justifiable. Certainly, some studies have found that high complication rates occur in emergency patients in whom operative intervention is deferred [11,12]. In addition, operative delays are often underestimated and anecdotal experience suggests that many deferred patients suffer significant ongoing delays following prioritisation of the following days emergency list. Hence, many surgeons feel that there are considerable arguments to be made in favour of prompt surgical treatment.

Trusts have also been adopting the Hospital At Night project and data from initial surveys of the project demonstrated that the amongst adult core specialities, General medicine has the highest number of calls and workload throughout the night followed by General Surgery which has half the number of calls [13]. The Academy of Royal Colleges supports a multidisciplinary approach to working at night as most problems at night were deemed to be 'medical' but opined that surgeons and anaesthetists will still be needed [14]. Our study has not been influenced by the Hospital at Night project but in the future there may be implications on the volume of operating that is performed at night.

The on-call surgical service of juniors in the United Kingdom has changed over the years to a predominantly shift-working pattern whilst surgical Consultants provide 24 hour cover at most NHS Trusts. The Surgical team at night at most Trusts consists of a resident Surgical Specialist Registrar with other junior doctors. Certain NHS Trusts have Surgical Specialist Registrars who are non-resident at night but are called in to assess, manage and operate on patients as appropriate. Few patients seen as an emergency need an operation within minutes and most surgical patients who require an operation do not need to be treated in the middle of the night. The decisions regarding timing for surgery is extremely crucial and is mostly made by the night surgical team in conjunction with the Consultant. The introduction of shift working amongst junior surgical staff – a pre-requisite for EWTD compliance in many NHS hospitals – demands a national review of the night-time operating policy. More specifically, the question of whether adequately rested personnel can be appropriately employed to undertake non-essential emergency work after midnight, is raised? To this end NCEPOD have advocated caution regarding unnecessary nighttime operating in the 2003 WOW II report and the authors of this document advocate urgent discussion on this issue between the Royal Colleges, the Department of Health and the British Medical Association. It must be noted however that currently most consultant surgeons do not work shifts. As such any increase in night-time emergency operating might result in decreased senior participation unless consultant working patterns change. The acceptability of a decrease in consultant-led emergency service provision requires consideration. Hence, opposing arguments can be made in favour of both deferring as well as proceeding with 'non-essential' nighttime operating. It is difficult to envisage that a universal solution to this problem will be applicable to all hospitals if the desirability of senior-led emergency service provision is maintained.

## Conclusion

Our study suggests that the provision of an emergency general surgical service should assume that some cases will need to go to theatre in the middle of the night. A significant proportion of these cases will require senior supervision.
